# Supplementary material for: Is stock market development sensitive to macroeconomic indicators? A fresh evidence using ARDL bounds testing approach
Source: PLoS One. 2022 Oct 19;17(10):e0275708. doi: 10.1371/journal.pone.0275708 (PMC9581411; doi:10.1371/journal.pone.0275708)
Supplement: Attachment — Submitted filename: 03 Responses to Reviewers.docx [file pone.0275708.s001.docx]

# RESPONSE TO REVIEWERS

# REVIEWER # 2

***(Additional text/changes are highlighted/marked with Dark Red in updated Manuscript file)***

1. ***Introduction***: Given there are studies examining macroeconomic factors of stock market development in Pakistan, particularly Shahbaz et al. (2016) with similar model specification, estimation technique and empirical findings, I cannot see any significant contribution in this study. Therefore, you should revise your study to provide a strong motivation on how your study is contribution to the existing body of knowledge

***Compliance****:	Although there are several studies addressing the same topic, but the current study is different from them by several aspects which provide a significant motivation and rationale to undertake this study. For example:*

i. *Past single-country studies conducted in the last two decades are either used an older data or based on very short time span. For example, Garcia and Liu (1999) used 16 years data, Yartey (2010) covers only 14 years period, Quartey and Gaddah (2007) study is based on 13 years data. In contrast, this study covers the broader time span of latest 40 years (1980-2019) to provide more updated and comprehensive results.*
ii. *Similarly, past studies on Pakistan economy provide evidence on one or two determinants for stock market development; hence, in Pakistan context, this area was under-researched. For example, Shahbaz et al. (2008) focused on only* ***Economic Growth*** *to test its impact on stock market development. Similarly, the studies of Abdul Malik and Amjad (2013) and Shahbaz et al. (2013) are based on single macroeconomic factor, i.e., Foreign Direct Investment, in relation to stock market development. Therefore, this deficiency in literature particularly in Pakistan context gives us a logical justification to provide evidence on multiple macroeconomic variables for the development of stock market of Pakistan.*
iii. *Insofar the study of Shahbaz et al. (2016) is concerned, our study is different from several aspects. For example, our methodology is partially different from them as they measured the causality between variables using VECM model and Granger causality approach, whereas our study doesn’t. Likewise, our study variables as well as empirical results are quite different from them; hence our study draw different conclusion and provide different recommendations on selected macroeconomic factors.*

----------------------------------------------------------------------------------------------------------------

2. Since your discussion on Pakistan include the development of COVID-19 in 2020 2021, I suggest you extend your analysis to 2020 or 2021 if data allow. Currently, your study ended in 2019, the discussion on stock market development in 2020 and 2021 became irrelevant. On the discussion of COVID-19, I would expect you research to capture the influence of COVID-19 on stock market development in your model.

***Compliance****:	The dataset utilized for this study was limited to 2019 because the year 2020 and 2021 witnessed the global pandemic of Covid-19 outbreak. It taken the Pakistan to an unprecedented position where economic and financial resources were drying up. Pakistan economy was shocked by this epidemic and its outbreak has caused substantial losses to several sectors and an enormous impact on the stock market. It abruptly wreaked havoc on the figures of imports and exports, savings and investments which ultimately led the nation towards strict lock-down, suspension of flight operations, and seal of cross-border trade, and ultimately, all these bring the increased uncertainty and volatility in stock market of Pakistan. Keeping in view all these situations, it was the view of the authors that including 2020 and 2021 would not give the true reflection of the returns from the stock market as well as the macroeconomic factors, that’s why we intentionally didn’t include 2020 and 2021 into our time period.*

----------------------------------------------------------------------------------------------------------------

3. ***Literature review***: On the literature review, you should have a brief theoretical review on how macroeconomic determinants influence stock market development. See Ho and Iyke (2017).

***Compliance****:	A brief theoretical review of past studies is included in the second paragraph of Literature Review section.*

----------------------------------------------------------------------------------------------------------------

4. Line 124, Ho is she.

***Compliance****:	Corrected (‘his’ is replaced with ‘her’)*

----------------------------------------------------------------------------------------------------------------

5. ***Methodology***:	Equations 1 and 2 are wrongly specified. Apart for the dependent variable (MC) being lagged as the independent variables, all the other independent variables are not lagged. This is the idea of ARDL.

***Compliance****:*

*The basic and standard equation of ARDL bounds testing is given below (as a screenshot). As you can see that dependent variable (in short- and long-run terms) is being lagged accordingly. Therefore, the equations 1 and 2 given in manuscript are correct. Furthermore, you can explore Ho (2019, pp. 328-329) and (Shahbaz et al., 2016, p. 83) to confirm that our ARDL econometric form is correctly specified.*

----------------------------------------------------------------------------------------------------------------

6. You should motivate why there is dummy variable in the equation. What is it for? Why it is required given the economic environment in Pakistan during your study period.

***Compliance****:	Text /discussion on dummy variable is updated in Section 3.2*

----------------------------------------------------------------------------------------------------------------

7. On economic growth, neither the source your cited nor any existing theories suggest that the relationship is negative (substitutes). Please verify.

***Compliance****:	On economic growth (in section 3.1), references of theoretical studies are added. Furthermore, existing theories suggest the positive relationship between stock market development and economic growth, therefore, relevant text is replaced in same paragraph.*

----------------------------------------------------------------------------------------------------------------

8. ***Results and Discussion***:	In addition to the main results, you should conduct robustness check in your analysis. Since the negative relationship between trade openness and stock market development is against our conventional wisdom, you should clearly explain why trade openness negatively affects stock market development with possible theoretical explanation in the context of Pakistan. Also, the concept of trade and foreign portfolio investment should not be mixed in your discussion.

***Compliance****:*

- *Robustness tests are performed and summarized in Table 8.*
- *An adequate explanation / justification about the negative relationship between trade openness and stock market development is provided at the end of Section 4.3*
- *Finally, the concept of foreign portfolio investment has removed while discussing the trade openness as it really seems to be irrelevant here.*

----------------------------------------------------------------------------------------------------------------

***REFERENCES***

Abdul Malik, I., & Amjad, S. (2013). Foreign direct investment and stock market development in Pakistan. *Journal of International Trade Law and Policy*, *12*(3), 226-242. <https://doi.org/10.1108/JITLP-02-2013-0002>

Garcia, V. F., & Liu, L. (1999). Macroeconomic Determinants of Stock Market Development. *Journal of Applied Economics*, *2*(1), 29-59. <https://doi.org/10.1080/15140326.1999.12040532>

Ho, S.-Y. (2019). Macroeconomic determinants of stock market development in South Africa. *International Journal of Emerging Markets*, *14*(2), 322-342. <https://doi.org/10.1108/IJoEM-09-2017-0341>

Quartey, P., & Gaddah, M. (2007). Long Run Determinants of Stock Market Development in Ghana. *Journal of African Business*, *8*(2), 105-125. <https://doi.org/10.1300/J156v08n02_07>

Shahbaz, M., Ahmed, N., & Ali, L. (2008). Stock market development and economic growth: ARDL causality in Pakistan. *International Research Journal of Finance and Economics*, *14*(1), 182-195.

Shahbaz, M., Hooi Lean, H., & Kalim, R. (2013). The impact of foreign direct investment on stock market development: Evidence from Pakistan. *Economic research-Ekonomska istraživanja*, *26*(1), 17-32.

Shahbaz, M., Rehman, I. U., & Afza, T. (2016). Macroeconomic determinants of stock market capitalization in an emerging market: fresh evidence from cointegration with unknown structural breaks. *Macroeconomics and Finance in Emerging Market Economies*, *9*(1), 75-99. <https://doi.org/10.1080/17520843.2015.1053820>

Yartey, C. A. (2010). The institutional and macroeconomic determinants of stock market development in emerging economies. *Applied Financial Economics*, *20*(21), 1615-1625. <https://doi.org/10.1080/09603107.2010.522519>
